# Bayesian analysis of dynamic phosphoproteomic data identifies protein kinases mediating GPCR responses

**DOI:** 10.1186/s12964-022-00892-6

**Published:** 2022-06-03

**Authors:** Kirby T. Leo, Chung-Lin Chou, Chin-Rang Yang, Euijung Park, Viswanathan Raghuram, Mark A. Knepper

**Affiliations:** Epithelial Systems Biology Laboratory, Systems Biology Center, National Heart, Lung, and Blood Institute, National Institutes of Health, Bethesda, MD USA

**Keywords:** Protein kinase, V2 vasopressin receptor, Collecting duct, Bayes’ Theorem, Phosphoproteomics

## Abstract

**Background:**

A major goal in the discovery of cellular signaling networks is to identify regulated phosphorylation sites (“phosphosites”) and map them to the responsible protein kinases. The V2 vasopressin receptor is a G-protein coupled receptor (GPCR) that is responsible for regulation of renal water excretion through control of aquaporin-2-mediated osmotic water transport in kidney collecting duct cells. Genome editing experiments have demonstrated that virtually all vasopressin-triggered phosphorylation changes are dependent on protein kinase A (PKA), but events downstream from PKA are still obscure.

**Methods:**

Here, we used: 1) Tandem mass tag-based quantitative phosphoproteomics to experimentally track phosphorylation changes over time in native collecting ducts isolated from rat kidneys; 2) a clustering algorithm to classify time course data based on abundance changes and the amino acid sequences surrounding the phosphosites; and 3) Bayes’ Theorem to integrate the dynamic phosphorylation data with multiple prior “omic” data sets covering expression, subcellular location, known kinase activity, and characteristic surrounding sequences to identify a set of protein kinases that are regulated secondary to PKA activation.

**Results:**

Phosphoproteomic studies revealed 185 phosphosites regulated by vasopressin over 15 min. The resulting groups from the cluster algorithm were integrated with Bayes’ Theorem to produce corresponding ranked lists of kinases likely responsible for each group. The top kinases establish three PKA-dependent protein kinase modules whose regulation mediate the physiological effects of vasopressin at a cellular level. The three modules are 1) a pathway involving several Rho/Rac/Cdc42-dependent protein kinases that control actin cytoskeleton dynamics; 2) mitogen-activated protein kinase and cyclin-dependent kinase pathways that control cell proliferation; and 3) calcium/calmodulin-dependent signaling.

**Conclusions:**

Our findings identify a novel set of downstream small GTPase effectors and calcium/calmodulin-dependent kinases with potential roles in the regulation of water permeability through actin cytoskeleton rearrangement and aquaporin-2 trafficking. The proposed signaling network provides a stronger hypothesis for the kinases mediating V2 vasopressin receptor responses, encouraging future targeted examination via reductionist approaches. Furthermore, the Bayesian analysis described here provides a template for investigating signaling via other biological systems and GPCRs.

**Video abstract**

**Supplementary Information:**

The online version contains supplementary material available at 10.1186/s12964-022-00892-6.

## Background

A major goal in regulatory biology is to specify causal signaling models at a cellular level that connect external signals to functional responses. The chief elements of such models are proteins that undergo changes in abundance or changes in chemical state, e.g. due to post-translational modifications. Although many post-translational modifications contribute to signaling in eukaryotic cells, protein phosphorylation stands out as being the core process that carries the largest component of signaling information [1–3]. Consequently, signaling system identification depends strongly on measurement of changes in protein phosphorylation. A key tool in this quest is bottom-up quantitative protein mass spectrometry using phosphorylated tryptic peptides (phosphoproteomics) [2, 4]. Phosphoproteomics methodologies (and protein mass spectrometry methods in general) have undergone progressive improvements in sensitivity and precision over the past two decades allowing more and more complete descriptions of signaling networks. Here, we use a robust model system, vasopressin-dependent regulation of aquaporin-2 (AQP2)-mediated water transport in renal collecting duct cells [5–10], to investigate signaling downstream from the V2 vasopressin receptor (V2R).

Vasopressin plays an essential role in body fluid homeostasis [8]. It is secreted by the posterior pituitary gland in response to high blood osmolality. Vasopressin binds to V2Rs in AQP2-expressing collecting duct cells in the kidney to regulate osmotic water transport. The regulation occurs as a result of effects on membrane trafficking to move AQP2 water channels to the apical plasma membrane [11–13] and also through effects to increase *Aqp2* gene transcription [14–16]. The V2R is a G-protein coupled receptor (GPCR) that signals through the heterotrimeric G-protein alpha subunit (G_α_s) to activate adenylyl cyclase type 6 [17, 18] and increase cyclic adenosine monophosphate (cAMP) levels [19] in collecting duct cells. Protein kinase A (PKA) appears to be the main cAMP effector in the collecting duct, mediating altered phosphorylation of multiple proteins [20], many of which are themselves protein kinases that phosphorylate their own sets of substrates [21]. One major target of PKA is AQP2 [20]. Understanding vasopressin signaling in collecting duct cells therefore requires that experimentally determined vasopressin-regulated protein kinases be mapped to experimentally determined vasopressin-regulated phosphorylation sites. In addition, changes in the phosphoproteome can also be attributed to vasopressin’s ability to regulate protein phosphatases, e.g. through PKA mediated phosphorylation of ARPP19/ENSA [22]. Most of the data gathered thus far represent steady-state responses to vasopressin after 15 min or more of vasopressin exposure [21–25]. Additional information needed for kinase/substrate mapping can, in principle, be obtained through observation of dynamic responses to vasopressin since the various kinases involved are likely to be activated at different time points after initiation of vasopressin exposure.

Here, we have measured the dynamic response, in native rat inner medullary collecting duct (IMCD) cell suspensions, to V2R-selective vasopressin analog desmopressin (dDAVP) using quantitative phosphoproteomics to follow phosphorylation changes from 1 to 15 min after dDAVP addition. This time-course information is combined with phosphorylation site motif information to classify the vasopressin-responsive target groups. To identify the likely responsible kinases for these target groups, we use Bayesian data integration methods [26–29] with prior data describing subcellular localization of kinases and substrates, transcriptomic and proteomic identification of protein kinases expressed in collecting duct cells, and experimentally determined sequence preferences of individual protein kinases. An online resource has been created for data sharing. The data and analysis identify the key protein kinases downstream from PKA activation in response to ligand binding to a G_α_s-coupled GPCR.

## Methods

### Inner medullary collecting duct (IMCD) suspensions

IMCD suspensions were prepared from pathogen‐free male Sprague–Dawley rats weighing 200–225 g in accordance with the NHLBI Animal Care and Use Committee protocol number H-0110R5 as described previously [30, 31]. Rats were treated with furosemide (0.5 mg per rat, i.p.) for 30 min before euthanization to wash out inner medullary hypertonicity and thus reduce osmotic shock to the tissue. Rat inner medullas from 13 rats were dissected out, minced, and digested for 90 min at 37 °C in an enzyme solution containing 2.5 mg/ml collagenase B and 2.5 mg/ml hyaluronidase dissolved in sucrose buffer (250 mM sucrose and 10 mM triethanolamine, pH 7.6). After digestion, the suspension was spun at low-speed (70 × *g*, 20 s, 3X) to enrich the IMCD fraction. In each centrifugation, the pellets were resuspended in bicarbonate buffer solution (118 mM NaCl, 25 mM NaHCO_3_, 5.5 mM glucose, 5 mM KCl, 4 mM Na_2_HPO_4_, 2 mM CaCl_2_, and 1.2 mM MgSO_4_, pH 7.4, equilibrated with 5% CO_2_/95% air). We studied the time-dependent phosphorylation of IMCD proteins following exposure to dDAVP, a vasopressin V2R analog (1 nM), or its vehicle at 37 °C in a pH- and temperature-controlled chamber. Tubules were collected at four time points (1, 2, 5, 15 min).

At the end of each incubation time, IMCDs were harvested by centrifugation (16,000 × g, 20 s). They were solubilized in 200 ul of lysis buffer containing 1.5% SDS and 1X HALT™ protease/phosphatase inhibitor cocktail (Thermo Scientific, Rockford, IL) in 100 mM triethylammonium bicarbonate (TEAB) buffer, pH 7.5. Cells were lysed by sonication (Misonix, model 3000, Newtown, CT) (intensity 3, 90 s) and homogenized with a QIAshredder (spun at 16, 000 X *g* for 2 min). Sample protein concentration was determined by a BCA protein assay (Pierce, Rockford, IL). This entire procedure was repeated on separate days to create a total of three replicates for each time point.

### Reduction, alkylation, and in-solution protease digestion

Cell lysates were reduced with 10 mM dithiothreitol in 100 mM TEAB for 1 h at room temperature, and then alkylated with 17 mM iodoacetamide for 1 h in the dark at room temperature. After that, six volumes of pre-chilled (-20 °C) acetone were added to precipitate proteins overnight at − 20 °C. The precipitated proteins were harvested by centrifugation at 8000 × g for 10 min at 4 °C. After removal of acetone, the precipitated protein samples were digested with Trypsin/LysC (Promega) (1:50 wt/wt.) in 100 mM TEAB overnight at 37 °C. The digested peptides were quantified using Pierce Quantitative Colorimetric Peptide Assay (Thermo Fisher Scientific), and stored at − 80 °C until the TMT labeling step. The above steps were repeated for three biological replicates.

### Tandem mass tag (TMT) labeling and fractionation

After thawing the peptide samples, equal amounts (400 ug) of peptides from each sample were taken and the volume was adjusted to 100 ul of 100 mM TEAB for labeling with TMT Isobaric Mass Tag (TMT11Plex, Thermo Fisher Scientific) following the manufacturer’s instructions. After labeling, all samples from the same replicate were combined and desalted using hydrophilic-lipophilic-balanced extraction cartridges (Oasis). To enhance phosphopeptide identification, the combined TMT-labeled samples were fractionated into 12 fractions using high pH reverse-phase chromatography (Agilent 1200 HPLC System). The fractionated samples were dried in a SpeedVac (Labconco) and stored in at − 80 °C until phosphopeptide enrichment.

### Phosphopeptide enrichment

From each fraction, 5% was collected in a separated tube for “total” proteomics and the remaining 95% was processed for “phospho” proteomics. We followed the Sequential Enrichment from Metal Oxide Affinity Chromatography protocol from Thermo Fisher Scientific for the phosphopeptide enrichment. In brief, phosphopeptide enrichment was first processed with the High-Selected TiO_2_ kit (Thermo Fisher Scientific), and then the flow through was subsequently subjected to the High-Selected Fe-NTA kit (Thermo Fisher Scientific) per manufacturers’ instructions. The eluates from the two enrichments were combined, dried and stored at -80 °C until LC–MS/MS analysis.

### Liquid chromatography-tandem mass spectrometry (LC–MS/MS)

Total peptides and phospho-enriched peptides were reconstituted with 0.1% formic acid in LC–MS grade water (J.T. Baker) and analyzed using a Dionex UltiMate 3000 nano LC system connected to an Orbitrap Fusion Lumos mass spectrometer equipped with an EASY-Spray ion source (Thermo Fisher Scientific). Peptides were introduced into a peptide nanotrap at a flow rate of 5 μL/min. The trapped peptides were fractionated with a reversed-phase EASY-Spray PepMap column (C18, 75 μm × 50 cm) using a linear gradient of 4–32% acetonitrile in 0.1% formic acid (120 min at 0.3 μL/min). The default MS2 workflow was selected on the mass spectrometer for TMT quantification. The main settings for MS2 scan were as follows: HCD activation, 37% collision energy, 1.6 m/z isolated window, 50,000 Orbitrap resolution, AGC target of 50,000, and 120 ms maximum injection time.

### Mass spectrometry data processing and analysis

The raw mass spectra were searched against the rat UniProt reference proteome (UP000002494_10116.fasta, downloaded in August 2020) using MaxQuant 1.6.17.0, and lot-specific TMT isotopic impurity correction factors were used as recommended in the TMT product data sheets. “Trypsin/P” was set as the digestion enzyme with up to two missed cleavages allowed. Carbamidomethylation of cysteine (C) was configured as a fixed modification. Variable modifications included TMT labeling of lysine (K) or N-terminus, phosphorylation of serine, threonine and tyrosine (S, T, Y), oxidation of methionine (M). The false discovery rate was limited to 1% using the target-decoy algorithm. Other parameters were kept as the defaults. Results are reported as reporter ion intensity ratios between dDAVP-treated samples and vehicle controls with independent control observations for each dDAVP-treated sample. Control:control ratios were used to characterize the background variability of the method as described in Results. The mass spectrometry proteomics data have been deposited to the ProteomeXchange Consortium via the PRIDE [32] partner repository with the data identifier PXD031332.

### Time course clusters

Phosphorylation sites (“phosphosites”) showing significant changes were clustered into groups defined by the speed and direction of changes as well as the amino acid sequences surrounding the phosphorylated amino acid as detailed in Results. Post-translational modifications (PTMs) were analyzed with PTM-Logo (https://hpcwebapps.cit.nih.gov/PTMLogo/) from input amino acid sequences (13-mers) collected with PTM-Centralizer (https://esbl.nhlbi.nih.gov/PtmCentralizer/) [33]. The following representations were used to denote functionality: non-polar (Φ:AFILMVW), phosphorylatable polar (Δ:STY), acidic (Θ:DE), basic (Ψ:RKH), non-phosphorylatable polar (Σ:CNQ), proline (P), and glycine (G). Cluster group nomenclature is summarized in Additional file 1: Table S1.

### Bayesian analysis of protein kinases

Bayes’ Theorem was used to identify kinases most likely responsible for phosphorylating proteins in each time course cluster. Probabilities for candidate kinases are represented as a vector of length 521, corresponding to the 521 known protein kinases in the mammalian genome extracted from the UniProt/Swiss-Prot Protein Knowledgebase (https://www.uniprot.org/docs/pkinfam) [34]. The initial prior probability vector was assigned with equal probabilities for all elements (1/521) for an unbiased approach. We applied Bayes’ Theorem vector-wise seven successive times (Fig. 5A), each incorporating likelihood vectors representing a different experimental data set to stratify the 521 kinases with regards to probability of phosphorylating members of each phosphosite cluster. Python scripts and associated datasets are available at (https://github.com/krbyktl/time_course_bayes).

Calculations for each step are detailed in Additional file 2: Table S2. The first three steps assume that for protein kinases to phosphorylate proteins in the renal collecting duct, they must be expressed in collecting duct cells. Three large-scale expression datasets were selected and individually integrated as the first three Bayesian operations. RNA-seq data for microdissected IMCDs from Chen et al.[35] was selected to incorporate transcriptomic data for native IMCD cells while 15 min total protein expression data from the current paper and data from Limbutara et al.based on quantitative LC–MS/MS analysis of microdissected IMCDs [36] were used for the second and third steps of the Bayesian analysis. RNA-seq and LC–MS/MS datasets were translated into likelihood values with the use of the complement of the minimum Bayes’ factor (cMBF) [37]:

1 $$cMBF = 1 - e^{{\frac{{ - Z^{*2} }}{2}}}$$

Z^*^ represents the ratio of the integrated data value to the pivot parameter. The pivot parameter in the context of these expression data sets represents the intrinsic noise present in the experiment (Additional file 2: Table S2). Posterior probabilities for each step were calculated with the cMBF and priors using Bayes’ Rule:

2 $$P\left( {A{|}B} \right) = \frac{P(B|A) \cdot P\left( A \right)}{{P\left( B \right)}}$$

If a calculated likelihood value was smaller than 0.5, the value for that kinase was reset to 0.5 (a coin-flip probability) since zero values can potentially occur due to annotation problems or technical error for kinases. Such errors would wrongly eliminate a particular kinase and interfere with the overall robustness of the Bayesian analysis.

The fourth and seventh steps in the Bayesian analysis (Additional file 2: Table S2) accounts for differences in protein kinase target specificities utilizing data from Sugiyama et al.[38]. These authors identified target preference motifs for most protein kinases using incubation of proteins with recombinant kinases followed by mass spectrometry to identify phosphorylation events. For these data, substrate motifs were selected by filtering substrate UniProt IDs using parameters defined by Sugiyama et al., namely the presence of site-determining ions and localization probability (*P* > 0.75) based on PTM score. Selected substrate UniProt IDs were mapped to their corresponding sequence using the UniProt Retrieve/ID mapping tool (https://www.uniprot.org/uploadlists/). Detectable sequences from the ID search with valid phosphosites (i.e., an amino acid match with the reported position) were collected and converted into 13-amino-acid centralized sequences. “J” placeholders were created for overhang positions at the ends of proteins. A total of 151,394 centralized substrate sequences and their corresponding kinase matches were obtained (https://github.com/krbyktl/time_course_bayes/raw/master/data_files/filtered_kinase_substrates.xlsx).

For step 4 of the Bayesian analysis, we used the Sugiyama et al. data indicating preference of each kinase regarding the phosphorylated amino acid (S, T, or Y). Similarities in frequencies of S, T, and Y in the phosphorylated positions of the Sugiyama et al. substrate motifs and cluster motifs were ranked based on the dot-product of frequencies of S, T, and Y. Missing kinases among the Sugiyama et al. dataset were interpolated from the available data by assigning existing values for kinases to their closest KinMap neighbor (http://www.kinhub.org/kinmap/). The resulting dot-product scores for 479 kinases were used to calculate the cMBFs, and a pivot parameter of 1/9 was selected to reduce sensitivity between serine and threonine but maintain selectivity vis-a-vis tyrosine kinases. Thus, this Bayesian step (step 4) essentially selects kinases capable of phosphorylating S and/or T but not Y. Step 7, also based on the Sugiyama et al. data is discussed after steps 5 and 6.

For step 5, we matched fractionation data kinases versus putative substrates from a prior study on deep proteomic profiling [39] based on the assumption that a similar subcellular distribution between kinases and phosphosite clusters would indicate higher likelihoods of interaction. For proteins with multiple isoforms, the most abundant isoforms were used to represent protein distribution. A dot-product scoring of the abundance levels of these isoforms was used to match each cluster localization with the kinases’ localization to create 14 sets of unique rankings, which were integrated using Bayes’ Rule with a pivot parameter derived from the estimated rate parameter (l) from the assumed Poisson distribution of dot-product values (Additional file 2: Table S2).

For step 6 of the Bayesian analysis, we drew from literature searches on known kinases that change in activity in the collecting duct in response to vasopressin to find matches with the observed phosphorylation changes in clusters (Additional file 3: Table S3). Based on known kinase activation or inhibition, we classified notable kinases as decreasing, increasing, or ‘regulated with unknown activity change’ (indeterminate direction of activity) in response to vasopressin. If the net activity of a kinase matched the direction of the 15 min time point for a particular cluster, a cMBF of 0.9 was assigned. Otherwise, the kinases with non-matching denominations were assigned a cMBF of 0.1. If a kinase was labeled as ‘regulated with unknown activity change’ (defined above), a cMBF of 0.7 was assigned for all clusters. If no data existed for a kinase, the cMBF was assigned as 0.5. These cMBFs were incorporated into the pipeline with Bayes’ Rule to re-rank the posteriors from the colocalization integration.

For step 7, we again took advantage of the Sugiyama et al. dataset (see above) to match the amino acid sites surrounding the phosphosites between the preferred sequence from Sugiyama et al. and the actual sequences for each of the 14 phosphosite clusters. Selected position sites from kinase motifs in the Sugiyama et al. data were chosen based on the positions of the high frequency amino acids pinpointed during the initial clustering stages, e.g. + 1 for Group I, -3 and -2 for Group II.A. Observed frequencies of amino acids *a* in positions *i,* P(*a,i*), for each Sugiyama et al. substrate motif and cluster motif were transformed into information content (IC) scores for amino acids *a* at position *i* with Eq. (3) using the Human PhosphoSite Plus background [40] and entire amino acid sequence list of experimental 15 min timepoint phosphosites, respectively, as probabilities P_ref_(*a*) of finding an amino acid *a* in the background data.

3 $$IC\left( {a,i} \right) = P\left( {a,i} \right) \cdot log_{2} \left( {\frac{{P\left( {a,i} \right)}}{{P_{ref} \left( a \right)}}} \right)$$

Missing kinases were interpolated as detailed above for the phosphorylated position. These kinase and cluster information content scores were matched with a dot-product, which was then used to calculate the cMBFs. Pivot parameters were derived from the estimated rate parameter from the assumed Poisson distribution of the non-negative information scores. These cMBFs were incorporated into the Bayesian pipeline (step 7, Additional file 2: Table S2) to create final unique rankings of the kinases for each cluster (Additional File 4: Table S4).

### Network mapping

Top ranked kinases outlined in Fig. 5B were used to construct a model of vasopressin signaling. The top 5 hits for each group were combined and used to create a list of unique kinases likely to be responsible for vasopressin signaling. The identified kinases and other significant known molecules were related to each other through a literature screen to create “edges” of the network model (Additional file 5: Table S5). The direction, establishment of the relationship, and net target activity between “nodes” were collected to create the network in Cytoscape (Version 3.8.2). Non-ranked kinases or small molecules were only included in the network if they were important for explaining the relationships between top hits**.**

## Results

**Fig. 1:**
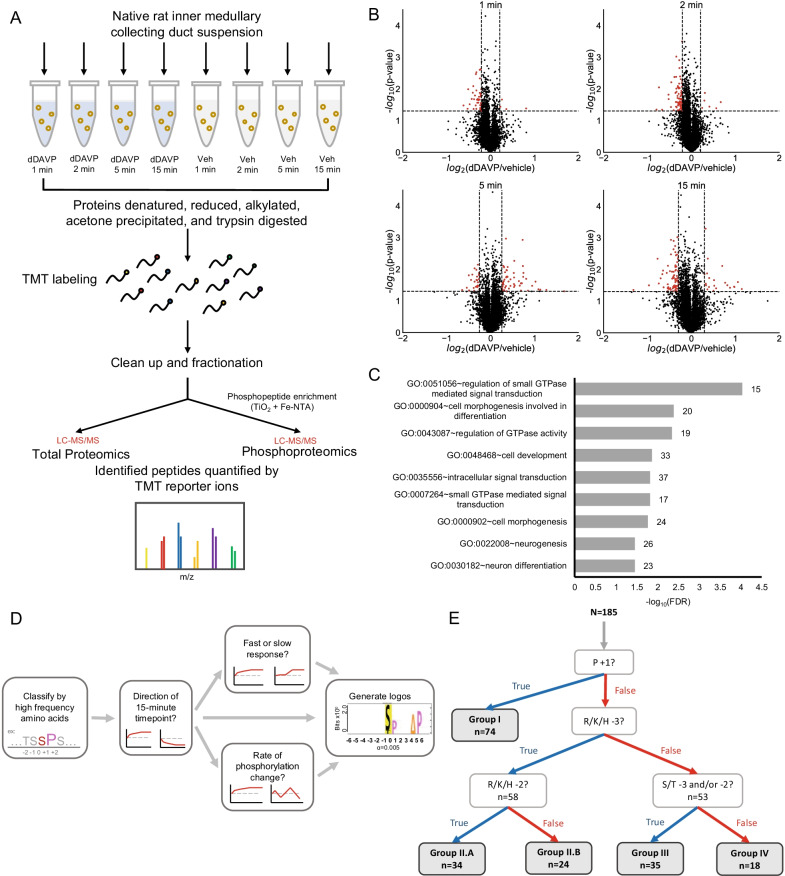
dDAVP stimulation protocol and phosphoproteomic data clustering. **A** Rat IMCDs were harvested and treated with dDAVP or vehicle for four time periods (1, 2, 5, 15 min). Three biological replicates were collected. Proteins were isolated, trypsinized, labeled with isobaric tags (TMT11plex), and fractionated. A portion of these peptides were set aside to determine total protein abundance in samples with the LC–MS/MS. The remaining peptides were enriched via High-Select TiO_2_ and Fe-NTA Enrichment Kits for LC–MS/MS. **B** Phosphoproteomic outputs (N > 6803) were visualized with volcano plots according to each time points’ statistical P-value and abundance (log_2_ of the treated and control sample ratio). Red points denote proteins significantly increased or decreased in phosphorylation. **C** Gene Ontology (GO) term analysis was performed with DAVID. Significantly enriched GO terms under the Biological Processes category were selected based on an FDR < 0.05. **D** Groups were characterized by amino acid sequence and time-course pattern. Groups of phosphosites were subclustered based on if they increased or decreased at the 15 min time point. The resulting subclusters were further subdivided via the speed and rate of change of phosphorylation. The subclusters were subsequently re-evaluated with PTM-Logo. **E** Phosphosites were clustered first by the highest positional frequency. From the remaining phosphosites, the next amino acid with the highest positional frequency of interest was assigned to Group II. This process was repeated once more to obtain Group III. The remaining phosphosites were assigned to Group IV

We carried out quantitative phosphoproteomic analysis of native rat IMCD suspensions exposed to the vasopressin analog dDAVP (1 nM) or vehicle for the following time periods: 1 min, 2 min, 5 min, and 15 min. Separate vehicle controls were collected at each time point, allowing each point to be analyzed with an independent statistical test. The TMT method was used for multiplexed quantification (Fig. 1A). In all, we quantified 6807 mono-phosphopeptides with distinct phosphorylation sites in 2690 phosphoproteins. Data for these phosphosites are provided as a data resource on a publicly accessible webpage (https://esbl.nhlbi.nih.gov/Databases/IMCD-TC/). We also quantified total protein abundances in 5444 proteins (https://esbl.nhlbi.nih.gov/Databases/IMCD-TC/total_data.xlsx) and filtered for proteins significantly altered in abundance (denoted by the “Outstanding” tab) but, in general, there were few changes at any of the observation times.

Among the phosphosites quantified, 185 exhibited significant changes based on dual criteria: a *p* < 0.05 (Student’s t-statistic) and the absolute value of log_2_(dDAVP/vehicle) outside of the 95% confidence range for one or more time points (Fig. 1B). The 95% confidence range is defined here based on the empirical Bayes’ method as the standard deviations of log_2_(dDAVP/vehicle) values across all 6807 observations for each time point. The standard deviation values were 0.104, 0.104, 0.128, 0.148 for the 1, 2, 5, and 15 min time points, respectively. Using the highest value among these, i.e. 0.148, the 95% confidence range is $[-\mathrm{0.30,0.3}0]$. Overall, the estimated false discovery rate is $0.05 \times 0.05=0.0025$, based on the dual criteria stated above.

The time courses for the 185 phosphosites that were changed can be viewed or downloaded at https://esbl.nhlbi.nih.gov/Databases/IMCD-TC/. Gene Ontology (GO) term analysis with DAVID was performed on these phosphoproteins, with the total protein abundance dataset serving as a reference background (Fig. 1C) [41]. “Regulation of small GTPase mediated signal transduction” stood out as the most significant Biological Process among other terms of morphogenesis and development. Next, the phosphosites were clustered and mapped to the protein kinases most likely to be responsible for phosphorylating all the sites within a cluster. Three criteria were used to identify clusters: 1) information content and frequencies of the amino acid sequence surrounding the phosphosites, 2) direction of change at the 15 min time point, and 3) relative rate of change in phosphorylation (Fig. 1D).

The strategy used started with the amino acid sequences surrounding the phosphosites. We performed first level clustering based on the position specific frequencies of the groups (Fig. 1E). The most frequent position specific cluster, Group I, was made up of peptides with a proline, P, in position + 1 relative to the phosphosite (n = 74 out of 185). Among the remaining 111 phosphosites, 58 had R,K, or H (represented by Ψ) in position -3 (Group II). Among the remaining 53 phosphosites, 35 had S, T, or Y (represented by Δ) in position -3 and/or -2 (Group III). Altogether, this first level clustering grouped 167 out of the 185 phosphosites. The remaining 18 phosphosites are classified here as Group IV. In the following paragraphs, we subcluster these preliminary clusters.

### Phosphosites with proline in position + 1

**Fig. 2:**
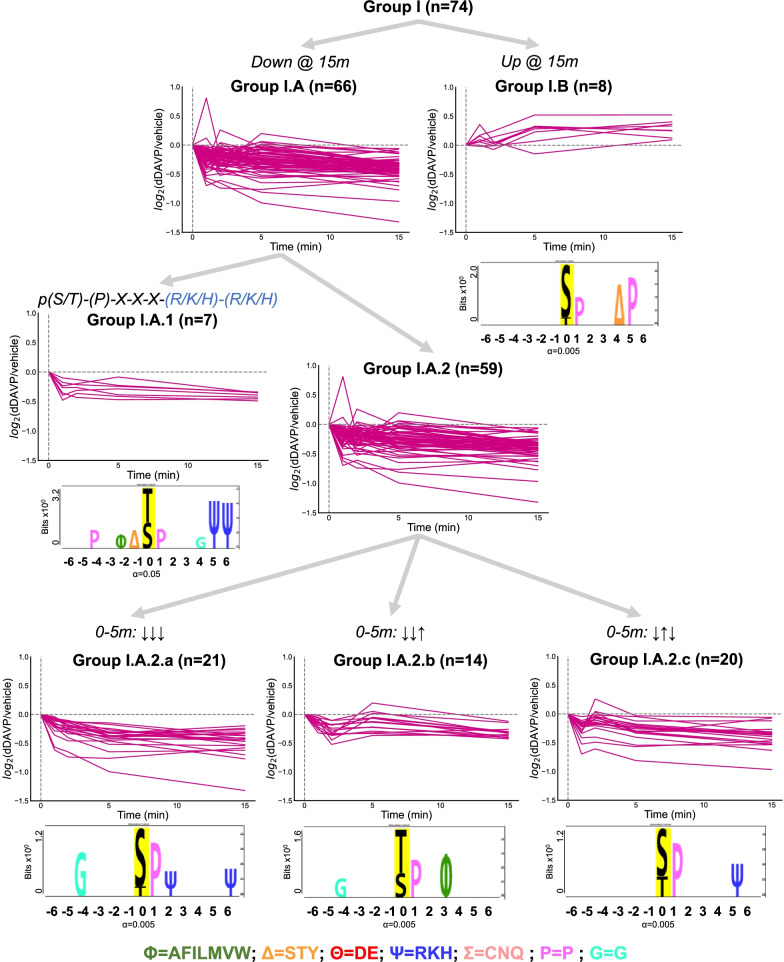
Clustering of proline-directed phosphosites: Group I. Logos were generated with PTM-Logo with a reference background of all 6807 peptides detected in our experiment. Representation of glycine in position − 4 in Group I.A.2.a was evaluated with respect to all 6807 phosphosites (n = 8) (Chi-square = 25.5; *p* < 0.00001). The incidence of a threonine as the targeted site for phosphorylation rather than a serine in Group I.A.2.b was evaluated similarly (n = 9) (Chi-square = 33.8; *p* < 0.00001). The following representations were used to denote functionality: non-polar (Φ:AFILMVW), phosphorylatable polar (Δ:STY), acidic (Θ:DE), basic (Ψ:RKH), non-phosphorylatable polar (Σ:CNQ), proline (P), and glycine (G)

The phosphosites with proline in + 1 (Group I) are typically phosphorylated by mitogen-activated protein kinases (MAPKs) or cyclin-dependent kinases (CDKs) [42]. The 74 phosphosites in this group could be readily separated into those that were decreased in abundance by vasopressin at the 15 min time point (n = 66, Group I.A) and those that were increased (n = 8, Group I.B), presumably indicative of different protein kinases being involved (Fig. 2). Seven of the 66 decreasing phosphosites had basic amino acids in positions + 5 and + 6 (as previously seen [21]) (Group I.A.1). The remaining 59 decreasing phosphosites (Group I.A.2) were partitioned into three groups based on their time-course pattern. One group (n = 21, Group I.A.2.a) showed a monotonic decrease over the 0–5 min period and had an overrepresentation of glycine in position -4. The second group (n = 14, Group I.A.2.b) had a complex time course with an initial decrease followed by an increase at the 5 min time point. This group had a predominance of threonine rather than serine as the phosphorylated amino acid. The third group (n = 20, Group I.A.2.c) also decreased initially but increased within 2 min. The complex pattern seen in the last two groups suggests that more than one kinase and/or phosphatase determined these time courses. The four remaining peptides with P in position + 1 could not be cleanly partitioned into any of the above groups.

Group I.B includes 8 phosphosites that, unlike most of the sites with P in + 1, increased in response to vasopressin. This suggests that some distinct MAP kinase or cyclin-dependent kinase is increased in activity. Mapping the sequences surrounding the individual phosphosites using PTM-Logo gave a unique sequence logo showing not only an S-P sequence in positions 0 and + 1, but also an S-P sequence in positions + 4 and + 5 (represented by Δ-P in Fig. 2). We discuss possible kinases that could phosphorylate peptides in this group below.

### Phosphosites with R, K, or H in position -3

**Fig. 3:**
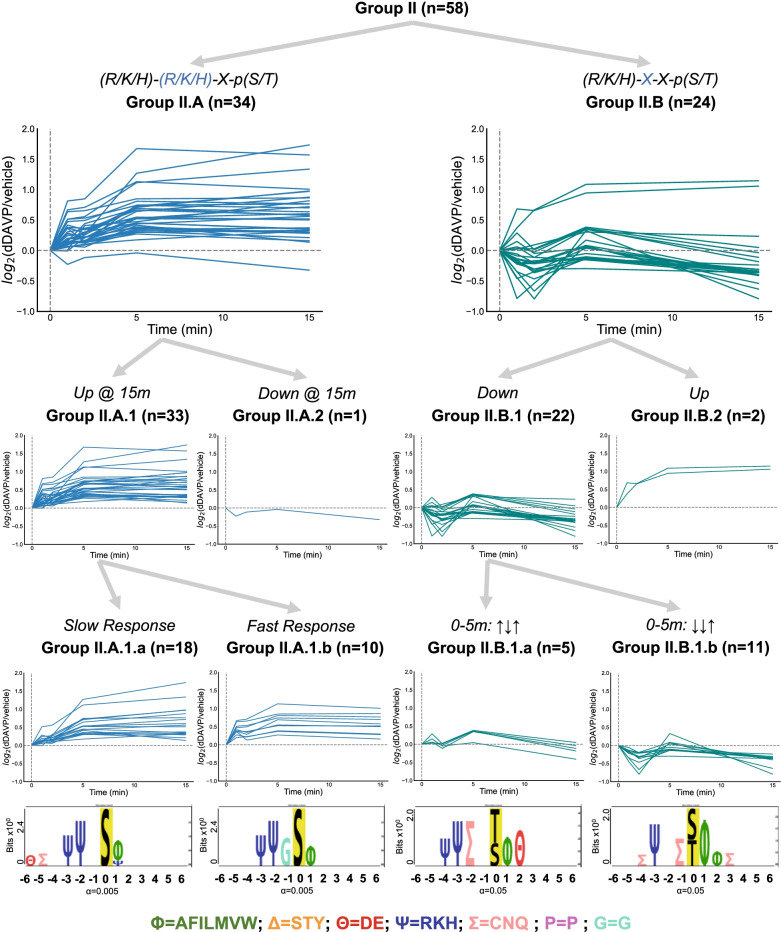
Clustering of phosphosites with upstream basic amino acids: Group II. Logos were generated with PTM-Logo with a reference background of all 6807 peptides detected in our experiment. A significant number of phosphosites in Group II.A were found to increase in abundance by 15 min when compared against all 185 regulated phosphosites (Chi-square = 7.3; *p* = 0.00695). Group II.A.1.b contained a significant number of sites with a glycine in the -1 position (n = 6) (Chi-square = 28.3; *p* < 0.00001 versus all 6807 mono-phosphopeptides)

The AGC and CAMK families are made up of so-called *basophilic* kinases that phosphorylate serines or threonines with basic amino acids (i.e., arginine, lysine, or histidine) upstream from the phosphosite. In general, we can identify these targets as the phosphosites with R, K, or H in position − 3 (Fig. 3, Group II**,** n = 58). These can be divided into those with R, K or H in position − 2 (Group II.A, n = 34) and those with other amino acids in this position (Group II.B, n = 24).

Those with R, K or H in both positions − 2 and − 3 are generally thought of as typical of sites phosphorylated by PKA or other similar basophilic kinases [1, 42, 43]. Among these, 33 of 34 of the time courses (Group II.A.1) showed increases at the 15 min time point, indicating that one or more basophilic kinases, including PKA, are activated by vasopressin. Most or all of these are likely to be phosphorylated by either of two PKA paralogs, i.e. PKA catalytic α or PKA catalytic β [1, 43]. Of the time courses that were increased, one cluster had a slow response, in which half of the maximum value response was not reached until 5 min or 15 min after vasopressin exposure (Group II.A.1.a, n = 18). Another group of the time courses clustered into those having a fast response, in which half of the maximum value response was reached within 1 min of vasopressin exposure (Group II.A.1.b, n = 10). This cluster had an over-representation of glycine, G, in the -1 position.

An additional 24 phosphosites had R, K, or H in position -3, but different (non-R, K, or H) amino acids in position -2 (Group II.B). This would be typical of basophilic protein kinases other than PKA, such as myosin light chain kinase (Mylk) and other calmodulin-dependent kinases [21, 44]. The time courses for these 24 phosphosites divide into two distinct temporal patterns. Two of the 24 showed progressive increases throughout the time course (Group II.B.2). The other 22 show a complex pattern that is ultimately decreased (Group II.B.1). The up-and-down pattern suggests that two or more regulatory factors target these sites with different time courses such as a protein kinase and a counteracting phosphatase. These phosphosites were clustered into two groups based on their time-course patterns, as done similarly for Group I.A.2. One group (n = 5, Group II.B.1.a) was characterized by an increase, then a decrease, before an increase prior to the five minute timepoint. The motif associated with this group aligns with a previously reported motif targeted by calcium/calmodulin-dependent protein kinase II delta and gamma (Camk2d and Camk2g) [1]. The other group decreased initially before increasing after the two minute timepoint (n = 11, Group II.B.1.b).

### Phosphosites with S, T, or Y in position -3 and/or -2

**Fig. 4:**
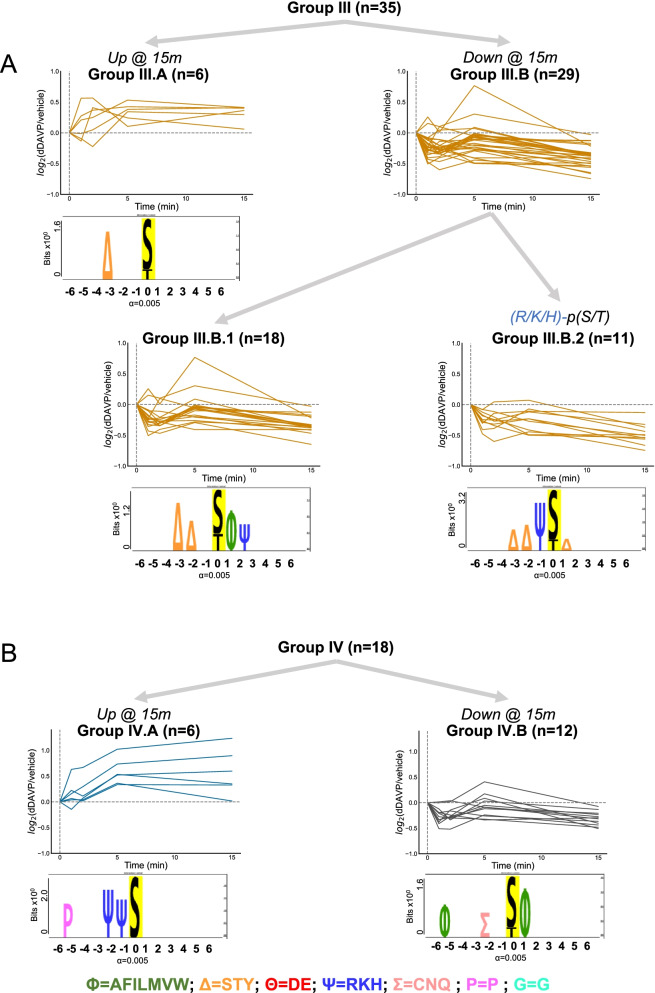
Clustering of atypical phosphosites. **A** Phosphosites with upstream phosphorylatable polar amino acids were clustered into Group III. This group had a significantly increased number of phosphosites decreased in abundance when contrasted with the 185 regulated phosphosites (Chi-square = 10.0; *p* = 0.001573). Compared with all 6807 mono-phosphopeptides, an increased number of phosphopeptides with an R, K, or H in position − 1 existed in Group III.B.2 (Chi-square = 13.8; *p* = 0.0002). Group III.B.1 alternatively had an increased presence of R, K, or H in position + 2 (Chi-square = 13.7, *p* = 0.0002). **B** Remaining phosphosites were categorized into Group IV

35 phosphosites were identified as containing a phosphorylatable polar amino acid (i.e. serine, threonine, or tyrosine) in positions -3 and/or -2 from the remaining phosphosites (Fig. 4A, Group III). 6 of these phosphosites were increased in abundance (Group III.A) while 29 were decreased (Group III.B). Within Group III.B, there was an overrepresented subset of phosphosites that contained an R, K, or H in position -1 (Group III.B.2, n = 11). The remaining phosphosites (Group III.B.1, n = 18) had an overrepresentation of R, K, or H in position + 2.

### Other phosphosites

The remaining unclassified phosphosites were designated into Group IV (Fig. 4B ,n= 18). 6 phosphosites were increased in abundance (Group IV.A) while 12 were decreased (Group IV.B). In Group IV.A, basic amino acids in positions -2 and -1 were highly represented. This overrepresentation was not seen in Group IV.B, which has few distinguishing motif characteristics.

### Bayesian analysis

**Fig. 5:**
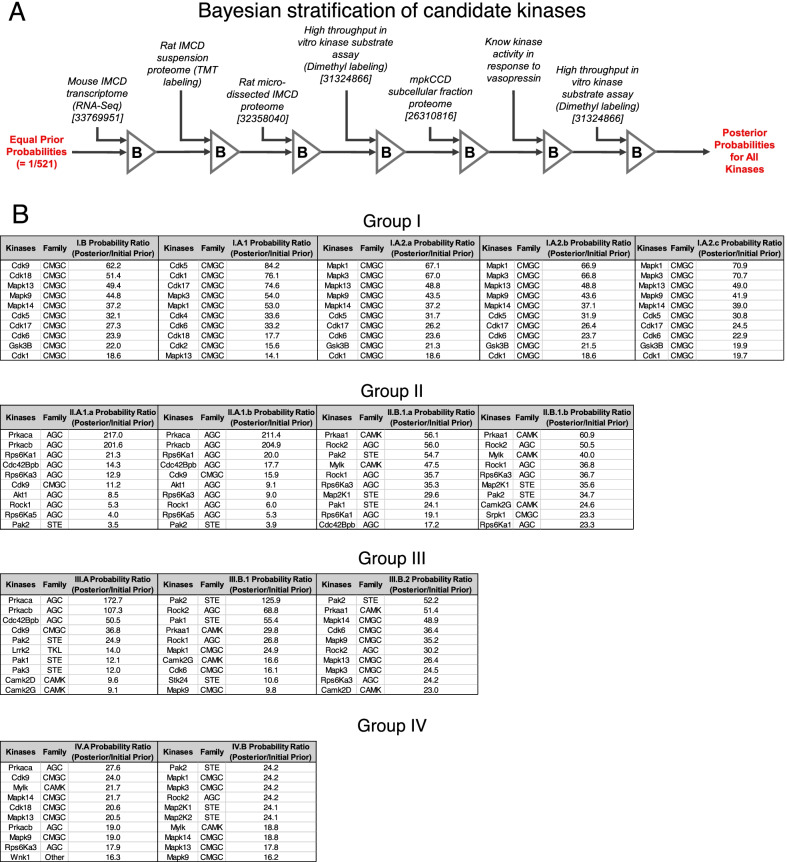
Bayesian stratification of candidate kinases. **A** 7 Bayesian operators representing different large-scale datasets were performed to rank 521 known kinases. Data sources and specific calculation method are included in Additional file 2: Table S2. **B** The top 10 kinases with the greatest likelihood of phosphorylating each cluster were reported as a probability ratio, with the initial prior representing the defined a priori 1/521 chance. Kinase family was reported with each corresponding kinase

With the majority of the 185 regulated phosphosites allocated into 14 clusters (Additional file 1: Table S1), we asked: which of the 521 known protein kinases are most likely responsible for phosphorylation changes in each cluster? This analysis assumes that members of each cluster, having similar properties, are likely to be phosphorylated by a common set of kinases. Because some of the clusters have overlapping properties, similar clusters may map to the same kinases especially since phosphatases can play a role in overall dynamics. We implemented an approach in which Bayes’ Theorem was applied to integrate multiple large-scale datasets (Additional file 2: Table S2) in order to identify the kinases most likely to map to each cluster (Fig. 5A). These datasets provide information about which kinases are expressed in collecting duct cells, colocalization of kinases and substrates, substrate sequence preferences, and prior data indicating regulation by vasopressin or cAMP-dependent signaling. These datasets were chosen with the intention to stratify the kinases with respect to their likelihoods. We used an unbiased approach which starts with equal prior probabilities (equivalent to 1/521 for each kinase).

**Fig. 6:**
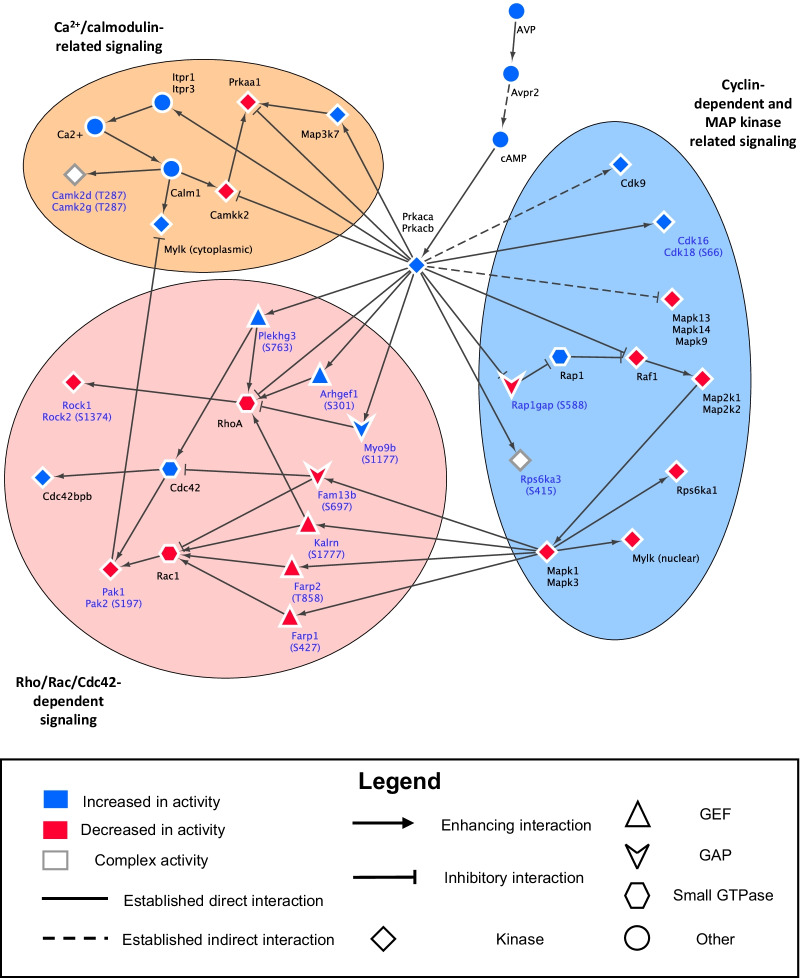
Causal mapping of ranked kinases. Kinases that were ranked top 5 for each cluster from the Bayes analysis were uniquely compiled into a causal map developed in Cytoscape. See Additional File 4: Table S4 for annotation of individual edges

We obtained 14 ranked lists of kinase gene symbols corresponding to the 14 clusters (Fig. 5B). Top ranked kinases in Group I primarily belonged to the CMGC kinase family, which are generally proline-directed serine/threonine kinases. Meanwhile, top ranked kinases in Group II mirrored the basophilic kinases, known to mainly fall within the AGC and CAMK kinase families, with some members in the CMGC and STE families [42]. Group III top ranked kinases took on a distribution of kinase families similar to that of the basophilic kinases. Ratios of the final posterior probability with respect to the initial equal prior probability were interpreted to correspond to the strength of the prediction of the kinase role. For example, protein kinase cAMP-activated catalytic subunit alpha and beta (Prkaca and Prkacb) in Group II.A.1.a ranked with a probability ratio of over 200 while all following kinases were ranked approximately tenfold lower. This indicates a similar, strong role for Prkaca and Prkacb for this group. Meanwhile, in Group II.B.1.b, the range of probability ratios for the top 10 kinases is smaller, and the confidence in the inference is relatively less than the previous example. The kinases that overall ranked at the top of the cluster lists were, in order: cyclin-dependent kinase 9 (Cdk9), cyclin-dependent kinase 5 (Cdk5), mitogen-activated protein kinase 1 (Mapk1), Prkaca, protein kinase AMP-activated catalytic subunit alpha 1 (Prkaa1), and P21 activated kinase 2 (Pak2). We used the information in Fig. 5B to construct a causal model of signaling in collecting duct cells (Fig. 6), described in the discussion.

## Discussion

Elucidating the black box of cellular signaling requires identification of key active enzymes and determining the order in which they interact with each other and their multiple substrates. Prior studies rely on reductionist approaches from steady state observations; however, such studies are limited in resolving network-wide causality. This study presents a multiplexed phosphoproteomic analysis that tracks protein phosphorylation over time across the proteome in response to addition of vasopressin. The data are integrated with time course-based clustering and Bayes’ Theorem. The resulting ranked lists of most-likely-responsible kinases for each time course cluster are compiled to hypothesize a mechanistic model of vasopressin action in the collecting duct (Fig. 6). The major nodes and edges in this model are summarized in Additional file 5: Table S5, which combines the current findings with evidence from the prior literature to identify the main elements of vasopressin signaling downstream from the initial activation of PKA. The model in Fig. 6 includes three subnetworks that are informative with regard to the question of how vasopressin regulates transport in collecting duct cells.

Among the three subnetworks, perhaps the most illuminating attribute of vasopressin action to regulate aquaporin-2 trafficking is “Rho/Rac/Cdc42 mediated signaling”. A major element of vasopressin action in collecting duct cells is the induction of a dramatic rearrangement of the actin cytoskeleton, resulting in dissolution of the apical cortical actin network necessary for movement of AQP2-containing intracellular vesicles to the plasma membrane [45–51]. The three small GTPases, Rho, Rac1 and Cdc42 (and their paralogs), have well establish roles in actin dynamics [52–54] and the current identification of this subnetwork is consistent with vasopressin-mediated actin rearrangement in collecting duct cells. The key finding is the presence of several GTPase-activating proteins (GAPs) and guanine nucleotide exchange factors (GEFs) that undergo phosphorylation in response to vasopressin (Fig. 6). These GAPs and GEFs exhibit selectivity with regard to which of the three small GTPases they regulate (Additional file 5: Table S5). Rho-associated coiled-coil-containing protein kinase 1 (Rock1), Rho-associated coiled-coil-containing protein kinase 2 (Rock2), P21 activated kinase 1 (Pak1), Pak2, and Cdc42 binding protein kinase beta (Cdc42bpb) are the relevant protein kinases in this subnetwork, and these mapped to several Group II and Group III phosphosite clusters. This opens the door for future studies to identify the specific roles of each vasopressin-regulated GAP and GEF and their respective downstream kinases in AQP2-containing vesicle trafficking and cytoskeletal organization.

Another important vasopressin-signaling subnetwork is “Cyclin-dependent and MAP kinases”. These kinases play many roles in cellular regulation, but among the most important in the context of vasopressin signaling is regulation of cell cycle and cell proliferation. In normal collecting duct cells, vasopressin decreases MAPK activity [21, 25, 55], but in autosomal dominant polycystic kidney disease (the most common genetic cause of chronic kidney disease) vasopressin increases MAPK activity driving growth of the epithelial cysts that are central to the loss of renal function [56, 57]. While most attention has been paid to Mapk3 and Mapk1, vasopressin regulates several CDKs that play basic roles in cell cycle regulation [20–22] and regulation of transcriptional elongation [16].

The third vasopressin-signaling subnetwork identified in this study is associated with calcium signaling, “Calcium-calmodulin related signaling”. Vasopressin not only triggers increases in cellular cAMP levels in collecting duct cells [19], but also increases intracellular calcium [19, 58] by increasing the frequency of transient calcium spikes in collecting duct cells [59, 60]. This response is PKA-dependent and likely results from PKA mediated phosphorylation of the IP_3_ receptors, inositol 1,4,5-triphosphate receptor type 1 and 3, calcium release channels present in the endoplasmic reticulum [61, 62] (Fig. 6 and Additional file 5: Table S5). Prior studies have shown that calcium buffering with 1,2-bis(o-aminophenoxy)ethane-N,N,N′,N′-tetraacetic acid “BAPTA” and calmodulin inhibitors block the water permeability response to vasopressin in isolated perfused collecting ducts [59], supporting an essential role of this pathway. Given the finding that inhibitors of the calmodulin-dependent kinase and Mylk also block the water permeability response to vasopressin [45], it seems likely that the Calcium-Calmodulin subnetwork and the Rho/Rac/Cdc42 subnetwork are functionally intertwined in the regulation of AQP2 trafficking. Camk2d and Camk2g were also identified in this study as being downstream from PKA signaling and have been proposed to play key roles in the phosphorylation of aquaporin-2 [26, 63]. In addition, a potentially important protein kinase in vasopressin signaling is Prkaa1 [64–67]. It is indirectly regulated by calcium-calmodulin through calcium/calmodulin dependent protein kinase kinase 2 (Camkk2) and was ranked highly as a potential kinase responsible for phosphorylation of basophilic sites in clusters II.B.1.a and II.B.1.b, as well as cluster III.B.2, all with decreasing phosphorylation over time.

Overall, we have pieced together a causal map of the downstream components of the vasopressin pathway with a clustering process based on the time course of vasopressin responses and a consequent list of identified top ranked kinases. A portion of the work here validated previous knowledge on the significant role of increased PKA signaling and subsequent downstream targeting, especially in proline-directed kinases like Mapk1 and Mapk3, which are inhibited through PKA dependent phosphorylation events. A novel set of basophilic kinases with previously uncertain roles in vasopressin/PKA signaling in the renal collecting duct was identified, including Prkaa1, Rock1 and Rock2, Pak1 and Pak2, and Cdc42bpb. These kinases play likely roles in the regulation of actin cytoskeleton dynamics and AQP2 trafficking to increase water permeability. These kinases are downstream small GTPase effectors, and numerous direct phosphorylation targets from the experimental data collected were noted to be GTPase-activating proteins or guanine nucleotide exchange factors that regulate Rho Family GTPases. We anticipate that there may be more phosphosites that play a role in this signaling pathway and are not within the set of detectable, trypsin-digested peptides. Yet, these current findings describe a previously unelucidated signaling pathway that may partially explain the morphological changes that occur in response to vasopressin related to actin cytoskeleton remodeling. These novel elements of vasopressin signaling are prime targets for CRISPR-based knock out or mutation studies in both cell culture systems and animals [20, 67, 68]. In addition to identifying a network of signaling through V2R, the findings of the current study will quite likely prove informative for understanding signaling via G_α_s-adenylyl cyclase-linked GPCRs in general.

## Conclusions

We propose a signaling network for a model GPCR, the V2R in kidney, based on Bayesian analysis of dynamic phosphoproteomic data integrated with other large-scale “omics” data. The novel set of downstream small GTPase effectors and calcium/calmodulin-dependent kinases identified here elucidate signaling in the collecting duct and are prioritized targets for further reductionist studies. We emphasize the use of this methodology in resolving network-wide causality; the described Bayesian analysis should be used as a flexible framework for probing key kinases involved in GPCR signaling in other biological systems.

## Supplementary Information

**Additional file 1: Table S1.** Assignment of phosphosites to clusters. 185 phosphosites were clustered based on criteria defined in Results. Gene symbol and site information are provided. Time course data associated with each phosphosite is located at: https://esbl.nhlbi.nih.gov/Databases/IMCD-TC/.

**Additional file 2: Table S2.** Data and calculations for Bayesian analysis of the most likely kinases to phosphorylate each phosphosite cluster. Bayes' Theorem was applied seven successive times to incorporate various large-scale datasets through likelihood vectors. Sources for each dataset are listed as PMIDs and an explanation for usage is provided. Likelihood calculations were completed through the use of cMBFs with defined Pivot parameters.

**Additional file 3: Table S3.** Known net vasopressin effects on kinases. Literature searches on known kinases that change in activity in the collecting duct in response to vasopressin were performed. Identified kinases were classified as “Decrease”, “Increase”, or “Regulated” with regard to net effect on activity depending on the findings in these sources. This information was utilized for step 6 of the Bayesian analysis.

**Additional file 4: Table S4.** Ranking of all kinases in their likelihood of phosphorylating each phosphosite cluster. The final computed output from the Bayesian analysis was represented as probability ratios of Posterior/Initial Prior where the Initial Prior is the defined *a priori *1/521 chance.

**Additional file 5: Table S5.** Nodes and edges for the causal map of vasopressin effects in IMCD. Source nodes were mapped to Target nodes based on literature searches and data from this paper. Information from these sources defined whether the direction of the edge was “Enhancing” or “Inhibitory”, if the edge was based on a “Direct” or “Indirect” relationship, how to classify the Target node, and if the Target node “Increased”, “Decreased”, or had “Complex” activity within the network. Data were imported into Cytoscape (Version 3.8.2) to form the causal map described in Figure 6.

## Data Availability

The datasets used and analyzed during the current study are included within the article and its additional files. Data generated by the authors are available through the ProteomeXchange Consortium via the PRIDE [32] partner repository with the data identifier PXD031332 or at https://esbl.nhlbi.nih.gov/Databases/IMCD-TC/. The scripts, data used, and calculations for the Bayesian Analysis are in the “time_course_bayes” repository: https://github.com/krbyktl/time_course_bayes.

## Abbreviations

AQP2: Aquaporin-2
V2R: V2 vasopressin receptor
GPCR: G-protein coupled receptor
G_α_s: G-protein alpha subunit
cAMP: Cyclic adenosine monophosphate
PKA: Protein kinase A
IMCD: Inner medullary collecting duct
dDAVP: Desmopressin
TMT: Tandem mass tag
Phosphosites: Phosphorylation sites
PTM: Post-translational modification
cMBF: Complement of the minimum Bayes’ factor
IC: Information content
GO: Gene ontology
MAPK: Mitogen-activated protein kinase
CDK: Cyclin-dependent kinase
Mylk: Myosin light chain kinase
Camk2d: Ca^2+^/calmodulin-dependent protein kinase II delta
Camk2g: Ca^2+^/calmodulin-dependent protein kinase II gamma
Prkaca: Protein kinase cAMP-activated catalytic subunit alpha
Prkacb: Protein kinase cAMP-activated catalytic subunit beta
Cdk9: Cyclin-dependent kinase 9
Cdk5: Cyclin-dependent kinase 5
Prkaa1: Protein kinase AMP-activated catalytic subunit alpha 1
Pak2: P21 activated kinase 2
GAP: GTPase-activating protein
GEF: Guanine nucleotide exchange factor
Rock1: Rho-associated coiled-coil-containing protein kinase 1
Rock2: Rho-associated coiled-coil-containing protein kinase 2
Pak1: P21 activated kinase 1
Cdc42bpb: Cdc42 binding protein kinase beta
