# Supplementary material for: Bayesian analysis of dynamic phosphoproteomic data identifies protein kinases mediating GPCR responses
Source: Cell Commun Signal. 2022 Jun 3;20:80. doi: 10.1186/s12964-022-00892-6 (PMC9164474; doi:10.1186/s12964-022-00892-6)
Supplement: Supplementary file 2 — Additional file 1: Table S1. Assignment of phosphosites to clusters. 185 phosphosites were clustered based on criteria defined in Results. Gene symbol and site information are provided. Time course data associated with each phosphosite is located at: https://esbl.nhlbi.nih.gov/Databases/IMCD-TC/. [file 12964_2022_892_MOESM2_ESM.docx]

**Additional file 1: Table S1.** Assignment of phosphosites to clusters

| **Cluster** | **Phosphopeptides** |
| --- | --- |
| **I.B** | Usp32 (S1430), Pak6 (S347), Cttnbp2nl (S562), Gmip (S434), Gigyf2 (S26), Eps8l1 (S182), Erc1 (S17), Gsn (T557) |
| **I.A.1** | Ankhd1 (T1527), Kank1 (T1340), Camsap3 (S771), Cobll1 (T246), Fam13b (S697), Ppp1r37 (S581), Kif1c (T1077) |
| **I.A.2.a** | Apc (S2350), Ablim1 (S142), Cobll1 (T112), Rnf123 (S679), Lmtk2 (S1478), Ahnak (S2725), Farp1 (S427), Ahnak (S5330), Ppm1h (S220), Ppm1h (T223), Mark2 (S453), Plekha7 (S536), Ppp1r14a (S26), Ahnak (S2691), Ahnak (S4893), Ubxn6 (S119), Dis3l2 (S12), Septin9 (S67), Sptbn2 (S2192), Kalrn (S1777), Lasp1 (T104) |
| **I.A.2.b** | Rab11fip5 (T779), Efnb1 (S286), Nsd3 (S447), Stam (S156), Ralgapb (T363), Prkch (S678), Ahnak (T4744), Map3k11 (S525), Ahnak (T691), RGD1561149 (T530), Farp2 (T858), Ahnak (T547), Ahnak (T2603), Agap3 (T308) |
| **I.A.2.c** | Vcpip1 (S767), Triobp (S1607), Ppm1h (T113), Slco4a1 (S39), Klrg2 (S49), Sh2b1 (S219), Map4k4 (S760), Celsr1 (S2678), Ppl (S14), Map2 (T1625), Elk1 (S276), Ahnak (T716), Fam126b (T306), rCG37581 (S94), Anapc1 (S555), Pkn2 (S307), Tnks1bp1 (S1628), Tjp3 (T864), Septin9 (T107), Dennd4c (S1287) |
| **II.A.1.a** | Ccs (S267), Tbc1d12 (S202), Lrrfip1 (S88), RGD1561662 (S1134), Gys1 (S698), Ctnnb1 (S552), Rab11fip1 (S386), Atp11c (S1106), Myo9b (S1177), Mtm1 (S23), Stmn1 (S63), Kifap3 (S102), Apc (S2793), Aqp2 (S256), Slc44a3 (S590), Cgn (S332), Rps6ka3 (S415), Lrba (S1225) |
| **II.A.1.b** | Arhgef1 (S301), Plekhg3 (S763), Luzp1 (S261), Cdk18 (S66), Plxdc2 (S507), Kif13b (S1794), Pdlim5 (S228), Fam83h (S970), Lrrfip2 (S133), Pum1 (S709) |
| **II.B.1.a** | Tmsb4x (T23), Ralgapa1 (T753), Plec (T4033), R3hdm2 (S347), Dsp (S2216) |
| **II.B.1.b** | Camk2d (T287), Camk2g (T287), Grip1 (T956), Sptbn1 (T2322), Lmnb2 (T441), Itsn2 (S211), Pik3c2a (S340), Pxn (S279), RGD1561662 (S908), Arhgap27 (S181), Parva (S28) |
| **III.A** | Slc38a1 (S52), Uvrag (S549), Rap1gap (S588), Sh3bp5l (S342), Lad1 (S323), Mfsd5 (T442) |
| **III.B.1** | Pi4ka (S254), Utrn (S2208), Zc3h12c (S772), Crtc3 (S370), Arhgef16 (S9), Tln1 (T144), Eef2 (T57), Hspb1 (S180), Raph1 (S140), Sptbn1 (T2181), F11r (S297), Sh3gl1 (S288), Ahnak (S5417), Tmsb4x (T34), Pkp3 (S205), Trim28 (S502), Tpd52l1 (S165), Tex2 (T197) |
| **III.B.2** | Ap3d1 (S685), Farp1 (S373), Ahnak (T151), Prkd2 (S225), Nipal3 (S395), F11r (S288), Crtc3 (S329), Pak2 (S197), Dhrs13 (S347), Septin7 (T425), Proser2 (S413) |
| **IV.A** | Kalrn (S1772), Apc (S2792), Ajuba (S89), Srp14 (S44), Slc7a6os (S304), Dsp (S2214) |
| **IV.B** | Afdn (T1341), Raph1 (S138), Rock2 (S1374), Il10rb (S298), Usp53 (S47), Epb41l1 (S780), Ahnak (S4892), Pard3 (S1046), Cgn (S288), Zfyve16 (S934), Arvcf (T637), Leng1 (S59) |
