# Supplementary material for: Bayesian analysis of dynamic phosphoproteomic data identifies protein kinases mediating GPCR responses
Source: Cell Commun Signal. 2022 Jun 3;20:80. doi: 10.1186/s12964-022-00892-6 (PMC9164474; doi:10.1186/s12964-022-00892-6)
Supplement: Supplementary file 3 — Additional file 2: Table S2. Data and calculations for Bayesian analysis of the most likely kinases to phosphorylate each phosphosite cluster. Bayes' Theorem was applied seven successive times to incorporate various large-scale datasets through likelihood vectors. Sources for each dataset are listed as PMIDs and an explanation for usage is provided. Likelihood calculations were completed through the use of cMBFs with defined Pivot parameters. [file 12964_2022_892_MOESM3_ESM.docx]

**Additional File 2: Table S2**. Data and calculations for Bayesian analysis of the most likely kinases to phosphorylate each phosphosite cluster

| **Source** | **Dataset description** | **Explanation** | **Likelihood calculation** | **Pivot parameter** |
| --- | --- | --- | --- | --- |
| 33769951 | Mouse IMCD transcriptome (RNA-seq) | A kinase that phosphorylates peptides in IMCD must be expressed in IMCD | The complement of the minimum Bayes' factor $=1-e^{-\frac{Z^{*2}}{2}}$, where Z* is the ratio of the integrated data value to the pivot parameter. Minimum likelihood = 0.5 | TPM = 1 |
| This paper | Rat native IMCD suspension proteome | A kinase that phosphorylates sites in IMCD must be expressed in IMCD | The complement of the minimum Bayes' factor $=1-e^{-\frac{Z^{*2}}{2}}$, where Z* is the ratio of the integrated data value to the pivot parameter. Minimum likelihood = 0.5 | 5th percentile, Intensity = 158,296 |
| 32358040 | Rat micro-dissected native IMCD proteome | A kinase that phosphorylates sites in IMCD must be expressed in IMCD | The complement of the minimum Bayes' factor $=1-e^{-\frac{Z^{*2}}{2}}$, where Z* is the ratio of the integrated data value to the pivot parameter.  Minimum likelihood = 0.5 | 5th percentile, Copy per cell = 131 |
| 31324866 | Human high-throughput in vitro kinase substrate assay | Phosphosites with a S, T, or Y as their phosphorylated amino acid are more likely to be phosphorylated by kinases that target those amino acids | The complement of the minimum Bayes' factor $=1-e^{-\frac{Z^{*2}}{2}}$, where Z* is the ratio of the integrated data value to the pivot parameter.  Minimum likelihood = 0 | 0.11 |
| 26310816 | mpkCCD subcellular fraction proteome | Phosphosites in a certain subcellular fraction are more likely to be phosphorylated by kinases residing in the same subcellular fraction | The complement of the minimum Bayes' factor $=1-e^{-\frac{Z^{*2}}{2}}$, where Z* is the ratio of the integrated data value to the pivot parameter.  Minimum likelihood = 0 | Means of dot scores attributed to each cluster |
| See **Additional file 3:** **Table S3** | Compilation of known kinase activity in response to vasopressin | Kinases that increase in activity in response to vasopressin are more likely to be responsible for phosphosites that increase in phosphorylation | The integrated likelihood is assigned 0.9 for a match, 0.1 for nonmatch, 0.7 for kinases that have known phosphorylation changes due to vasopressin but in an indeterminate direction, and 0.5 for kinases with no annotation. | None |
| 31324866 | Human high-throughput in vitro kinase substrate assay | Phosphosites with a specific amino acid near the phosphorylated amino acid are more likely to be phosphorylated by kinases that are known to target motifs with those amino acids | The complement of the minimum Bayes' factor $=1-e^{-\frac{Z^{*2}}{2}}$, where Z* is the ratio of the integrated data value to the pivot parameter.  Minimum likelihood = 0 | Means of non-negative dot scores attributed to each cluster |
