# Supplementary material for: Bayesian analysis of dynamic phosphoproteomic data identifies protein kinases mediating GPCR responses
Source: Cell Commun Signal. 2022 Jun 3;20:80. doi: 10.1186/s12964-022-00892-6 (PMC9164474; doi:10.1186/s12964-022-00892-6)
Supplement: Supplementary file 4 — Additional file 3: Table S3. Known net vasopressin effects on kinases. Literature searches on known kinases that change in activity in the collecting duct in response to vasopressin were performed. Identified kinases were classified as “Decrease”, “Increase”, or “Regulated” with regard to net effect on activity depending on the findings in these sources. This information was utilized for step 6 of the Bayesian analysis. [file 12964_2022_892_MOESM4_ESM.docx]

**Additional file 3: Table S3**. Known net vasopressin effects on kinases

| **Kinases** | **Net Effect on Activity** | **Source (PMID)** |
| --- | --- | --- |
| ROCK2 | Decrease | This paper 17446864 11278652 |
| PAK2 | Decrease | This paper 26239621 16574378 21098037 |
| PAK1 | Decrease | 26239621 16574378 21098037 |
| CAMKK2, SIK2 | Decrease | 33346914 |
| ARAF, RAF1 | Decrease | 31313956 |
| MAP3K5 | Decrease | 28973931 |
| MAPK3, MAPK1, MAP2K1, MAP2K2 | Decrease | 18667481 |
| ROCK1 | Decrease | 17446864 11278652 |
| AKT1, AKT2, AKT3 | Increase | 18667481 |
| PRKD2 | Increase | 33346914 |
| CDK18, CDK16, PRKD1, SRC | Increase | 31313956 |
| PRKACA, PRKACB, WNK1, MTOR | Increase | 28973931 |
| ATM, CDK9, EIF2AK2, CDK7 | Increase | 22440904 |
| CAMK2G, CAMK2D | Regulated | This paper 11931644 26310817 24598363 |
| MAP4K4, PAK6, PKN1, RPS6KA3 | Regulated | This paper |
| MAPK11, MAPK12, MAPK13, MAPK14 | Regulated | 18667481 20724536 24556353 |
| MAPK10, MAPK8, MAPK9 | Regulated | 18667481 20139300 |
| AAK1, CSNK1D, MYLK, ERBB2, LMTK2, MAP3K11, MAP3K2, MAP3K3, MAP4K5, MARK3, NEK4, NRBP2, PEAK1, SCYL2, SRPK1, TAOK1 | Regulated | 33346914 |
| ERBB3, KALRN, MAP3K7, MARK2, MINK1, PRKCE, PRKCH, PRPF4B, PTK2 | Regulated | 31313956 |
